# Demographics and Clinicopathologic Profile of Pulmonary Sarcomatoid Carcinoma with Survival Analysis and Genomic Landscape

**DOI:** 10.3390/cancers15092469

**Published:** 2023-04-26

**Authors:** Asad Ullah, Asim Ahmed, Abdul Qahar Khan Yasinzai, Kue Tylor Lee, Israr Khan, Bina Asif, Imran Khan, Bisma Tareen, Kaleemullah Kakar, Gul Andam, Saleh Heneidi, Jaffar Khan, Hina Khan, Nabin R. Karki, Jaydira Del Rivero, Nagla Abdel Karim

**Affiliations:** 1 Department of Pathology, Microbiology, and Immunology, Vanderbilt University, Nashville, TN 37232, USA; 2 Medical College of Georgia, Augusta, GA 30912, USA; 3 Department of Medicine, Bolan Medical College, Quetta 83700, Pakistandrkaleemkakar@gmail.com (K.K.); gulsaleem765@gmail.com (G.A.); 4 Hackensack Meridian Health, Palisades Medical Center, North Bergen, NJ 07047, USA; 5 Bannu Medical College, Bannu 28100, Pakistan; 6 Department of Pathology, Cedars Sinai Medical Center, Los Angeles, CA 90048, USA; 7 Department of Pathology, Indiana University School of Medicine, Indianapolis, IN 46202, USA; 8 Division of Hematology and Oncology, Warren Alpert Medical School of Brown University, Providence, RI 02912, USA; 9 Mitchell Cancer Institute, University of South Alabama, Mobile, AL 36604, USA; 10 National Cancer Institute (NCI), Bethesda, MD 20892, USA; 11 Inova Schar Cancer Institute, University of Virginia, Fairfax, VA 22031, USA

**Keywords:** pulmonary sarcomatoid carcinoma, SEER, COSMIC, survival, genetics

## Abstract

**Simple Summary:**

Pulmonary sarcomatoid carcinoma (PSC) is a rare subtype of non-small cell lung carcinoma (NSCLC). In our study, we analyzed 5259 total cases of PSC, illuminating demographic trends and outcomes related to different treatment strategies. PSC mostly affects Caucasian males between 70 and 79. Male gender and distant spread were associated with poor clinical outcomes. Treatment with surgery was associated with better survival outcomes. The COSMIC analysis show the most common mutations in PSC are TP53, ARID1A, NF1, SMARCA4, and KMT2D. With this analysis, we hope to provide further data to better inform clinicians of effective treatment strategies for their patients.

**Abstract:**

**Background:** Pulmonary sarcomatoid carcinoma (PSC) is a rare subtype of non-small cell lung cancer (NSCLC) with an aggressive clinical nature and poor prognosis. With novel targeted therapeutics being developed, new ways to effectively treat PSC are emerging. In this study, we analyze demographics, tumor characteristics, treatment modalities, and outcomes of PSC and genetic mutations in PSC. **Methods:** Data from the Surveillance, Epidemiology, and End Results (SEER) database were reviewed to analyze cases of pulmonary sarcomatoid carcinoma from 2000 to 2018. The molecular data with the most common mutations in PSC were extracted from the Catalogue Of Somatic Mutations in Cancer (COSMIC) database. **Results:** A total of 5259 patients with PSC were identified. Most patients were between 70 and 79 years of age (32.2%), male (59.1%), and Caucasian (83.7%). The male-to-female ratio was 1.45:1. Most tumors were between 1 and 7 cm in size (69.4%) and poorly differentiated (grade III) (72.9%). The overall 5-year survival was 15.6% (95% confidence interval (95% CI) = 14.4–16.9)), and the cause-specific 5-year survival was 19.7% (95% CI = 18.3–21.1). The five-year survival for those treated with each modality were as follows: chemotherapy, 19.9% (95% CI = 17.7–22.2); surgery, 41.7% (95% CI = 38.9–44.6); radiation, 19.1% (95% CI = 15.1–23.5); and multimodality therapy (surgery and chemoradiation), 24.8% (95% CI = 17.6–32.7). On multivariable analysis, age, male gender, distant stage, tumor size, bone metastasis, brain metastasis, and liver metastasis were associated with increased mortality, and chemotherapy and surgery were associated with reduced mortality (*p* < 0.001). The best survival outcomes were achieved with surgery. The most common mutations identified in COSMIC data were TP53 31%, ARID1A 23%, NF1 17%, SMARCA4 16%, and KMT2D 9%. **Conclusions:** PSC is a rare and aggressive subtype of NSCLC, usually affecting Caucasian males between 70 and 79. Male gender, older age, and distant spread were associated with poor clinical outcomes. Treatment with surgery was associated with better survival outcomes.

## 1. Introduction

Pulmonary sarcomatoid carcinoma (PSC) is a mixed-phenotype neoplasm containing epithelial and sarcomatous elements. Epithelial–mesenchymal transition (EMT) is believed to play a role in these neoplasms’ pathogenesis and aggressive clinical nature [1]. PSC, a sarcomatoid carcinoma of the lung, is a rare form of poorly differentiated non-small cell lung cancer (NSCLC) that makes up roughly 0.1–0.4% of pulmonary malignancies and has a strong predilection to affect elderly men who smoke [2,3]. Smoking leads to damage of the respiratory epithelium, causing cellular instability and increasing the likelihood of subsequent carcinogenesis. Another important risk factor for pulmonary malignancy is radon exposure. Radon, a radioactive gas that is a decay product of uranium-238, releases alpha particles that damage the respiratory epithelium, generating oxidative stress and various cytotoxic effects that contribute to malignancy [4]. While radon is a well-known risk factor for lung malignancy, its association with PSC specifically is not well-studied. 

Our understanding of this cancer is limited, as accurate diagnosis remains a challenge. There are three main subvariants of PSC, each with characteristic histologies, pathologies, and molecular markers. These include pleomorphic carcinoma, carcinosarcoma, and pulmonary blastoma, with pleomorphic carcinoma being the most prevalent [5,6]. Pleomorphic carcinoma is further divided into spindle cell carcinoma and giant cell carcinoma subtypes. While many of these possess unique underlying pathology, there are shared characteristics between these five histological variants, determined by the shared role of EMT in their pathogenesis. PSCs generally present in central or peripheral locations of the upper lobes, and the presenting symptoms are non-specific and related to the involved structures [3,5]. Even though PSC is largely diagnosed by morphology, immunohistochemistry (IHC) is performed to differentiate PSC from other lung malignancies, such as malignant melanoma or mesothelioma, as well as to characterize the exact histological PSC subvariant [7,8]. Certain PSC subvariants stain similarly with cytokeratins, epithelial membrane antigen (EMA), and carcinoembryonic antigen (CEA), aiding clinicians in differentiating PSC from other lung malignancies. Sarcomatous components of PSC subvariants also possess unique staining patterns, aiding in further differentiation [3,6]. The sarcomatous components of PSC are theorized to be attributed to EMT, and this is driven by *KRAS/EGFR/P53* mutations, c-*MET* gene alterations, and overexpression of ZEB1, amongst many others. Specifically, *MET* exon 14 alterations have been observed to be more common in PSC than any other NSCLC subtypes. These mutations have been attributed to increased EMT and resistance to therapies such as tyrosine-kinase inhibitors (TKIs) and chemotherapy [3,6,9]. The median age of diagnosis of PSC is 65 years and the ratio of male to female patients is 4:1. Pulmonary blastomas tend to affect younger patients. 

PSC is currently managed as a conventional NSCLC. However, the aggressive clinical nature and advanced stage at initial diagnosis contributes to poor overall survival in PSC [3,5]. Surgery is the preferred treatment modality for patients diagnosed at early stages, often combined with adjuvant/neoadjuvant therapy. Despite PSC being resistant to chemotherapy and radiotherapy, the most common first-line chemotherapies are pemetrexed, paclitaxel, docetaxel, gemcitabine, and vinorelbine [10,11]. Use of targeted therapies and immune-checkpoint inhibitors is evolving as we gain more understanding of the molecular and biological features of this rare disease.

In this study, we summarized key points of PSC’s epidemiology, pathophysiology, diagnostic criteria, and current treatment regimens. In this study, we assessed demographic features of patients diagnosed with PSC and tumor characteristics. The demographic features incorporated in this analysis include age, race, and gender. We then conducted a survival analysis based on these demographic features and tumor characteristics. We also determined the demographic and tumor characteristics that are associated with poor prognosis.

## 2. Materials and Methods

We utilized the Surveillance, Epidemiology, and End Results (SEER) database, which was initiated in 1972 by the National Cancer Institute (NIH) and covers approximately 28% of the USA. Using the SEER*Stat software version (8.4.0), we collected data from 2000 to 2018 using International Classification of Diseases version 3 (ICD-O-3) codes. The data were exported to Statistical Analysis System (SAS) (SAS/ACCESS^®^ 9.4 Interface to ADABAS: Reference, SAS Institute Inc. 2013, Cary, NC, USA) for analysis.

The demographic data that we analyzed included age, race, and gender. The clinical data that we analyzed included tumor grade, tumor size, lymph node metastasis, treatment modality, and overall survival, including survival stratified by treatment modality. Every case in our study was previously confirmed genetically or histologically. The “type of reporting resource” for our cases included hospital facility, hospital/private laboratory, physician’s office/private medical practitioner, and nursing/convalescent home/hospice. The cases that were excluded from our analysis were those diagnosed by direct visualization without microscopic confirmation, radiography without microscopic confirmation, and clinical diagnosis only. For survival analysis, we only analyzed cases where the patient’s age was known, the case was confirmed microscopically, and the tumor presented with malignant behavior. The cases excluded from our survival analysis were cases diagnosed by death certificate only, autopsy only, and those alive with no survival time.

This study used Kaplan–Meier survival analysis and Cox regression to identify independent factors that affect survival. Missing or unidentified data were removed from multivariate analysis. Univariate analysis screened for significant factors to feed the multivariate model, and multivariate Cox regression analysis was then used to analyze the data. Statistical significance was defined as *p* < 0.05.

## 3. Results

In total, 5259 cases of PSC were identified from 2000 to 2018 in the SEER database. The molecular data with common mutations in PSC were extracted from the Catalogue Of Somatic Mutations in Cancer (COSMIC) database.

### 3.1. Demographic Characteristics and Tumor Characteristics: Grading, Staging, and Size

The mean age was found to be 69.2 years, with a standard deviation (SD) of ±11.5 years. Our analysis shows that PSC has a strong predilection for elderly patients, with over half of the cases occurring in patients 70 years or older and another 40% occurring in patients between 50 and 69 years. Gender-wise, we found that well over half (59.1%) of the cases occurred in males. The vast majority of cases occurred in white patients, followed by black, Asian or Pacific Islander, and American Indian or Alaskan Native.

The grading of PSC was known in over half of the total cases, and over 90% of these cases were classified as either poorly differentiated (Grade III) or undifferentiated/anaplastic (Grade IV). Well-differentiated (Grade I) and moderately differentiated (Grade II) cases were exceedingly rare, occurring in less than 4% of patients.

Our analysis organized the stages into distinct categories: localized, regional, distant, and unstaged/unknown. The localized stage indicates that the tumor was confined to the organ of origin without additional metastasis. It indicates infiltration beyond the basement membrane into the stroma. The regional stage indicates dissemination beyond the organ of origin and can be further subdivided according to direct extension and lymph node involvement. The distant stage indicates that the tumor or its cells spread beyond the primary site of origin to a non-local, non-regional area. The unknown stage refers to cases without sufficient evidence to accurately stage (https://training.seer.cancer.gov/staging/systems/summary/regionalized.html, accessed on 22 February 2023). Over 90% of the tumors were staged, and of the staged tumors, over half were staged as distant, followed by regional, then localized.

PSC size was known in over half of the total cases, and when known, overall size was relatively evenly distributed between 1–3 cm, 3.1–5 cm, 5.1–7 cm, and greater than 7 cm, with slight variation in each category. Greater than 7 cm was the most common size, followed by 3.1–5 cm, 1–3 cm, and 5.1–7 cm (Table 1).

### 3.2. Metastatic and Lymph Node Status at Diagnosis and PSC Treatment Characteristics

The lymph node status was unknown in 3397 (64.6%) cases and known in 1862 (35.4%) cases. Where known, 682 (36.6%) cases were positive while 1180 (63.4%) were negative.

**Figure 1:**
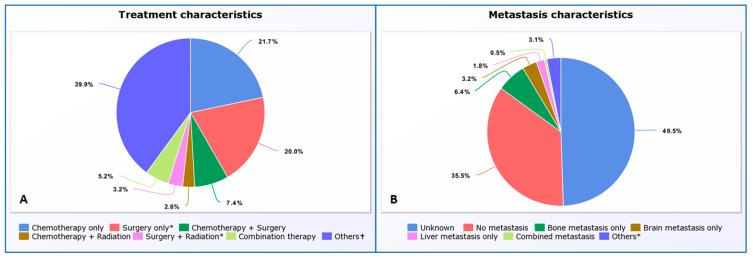
Pie Chart of Treatment Patterns (**A**) in PSC carcinoma and Metastasis patterns at the time of diagnosis (**B**); “other (* & †)” in (**A**,**B**) includes many different categories where the status of one of the treatments and pattern of metastasis are unknown.

Information on metastases was unknown in 2603 (49.5%) cases and known in 2656 (50.5%) cases. There were no metastases in 1869 (70.4%) of the known cases. Individual metastases in bone, brain, or liver were found in 338 (12.7%), 168 (6.3%), and 95 (3.6%) of the known cases, respectively, at the time of diagnosis. Bone and brain metastases combined were found in 62 (2.3%) cases, bone and liver metastases combined were found in 74 (2.8%) cases, and brain and liver metastases combined were found in 24 (0.9%) cases. Bone, brain, and liver metastases combined were found in 26 (1.0%) cases (Figure 1A and Supplementary Table S1).

Of the total cases, 1141 (21.7%) cases were treated with chemotherapy only, while 135 (2.6%) cases were treated with chemotherapy plus radiation and 387 (7.4%) cases with chemotherapy plus surgery. Of the total cases, 272 (5.2%) cases underwent combination (chemotherapy plus radiation plus surgery) therapy. In 1988 (37.8%) cases, the status of chemotherapy was unknown, and these cases underwent neither radiation nor surgery. Of the total cases, 2428 (2.2%) cases underwent chemotherapy without radiation, while the statuses of cases undergoing surgery were unknown. In 1915 (1.7%) cases, the statuses of both chemotherapy and surgery were unknown and did not undergo radiation therapy. In 1051 (20.0%) cases, the status of chemotherapy was unknown, but the cases underwent surgery without radiation. In 166 (3.2%) cases, the status of chemotherapy was unknown, but the cases underwent both radiation and surgery. In 77 (1.5%) cases, the status of chemotherapy was unknown, but these cases underwent radiation without surgery. In 28 (0.5%) cases, the statuses of both chemotherapy and surgery were unknown, and they did not undergo radiation therapy. In 12 (0.2%) cases, chemotherapy was performed without radiation and the status of surgery was unknown. In two (0.0%) cases, both chemotherapy and radiation were carried out, while the status of surgery were unknown (Figure 1B and Supplementary Table S2).

## 4. Outcomes and Survival Analysis

### 4.1. Survival Analysis by Age, Race, Gender, Tumor Grade, Stage, and Size

**Figure 2:**
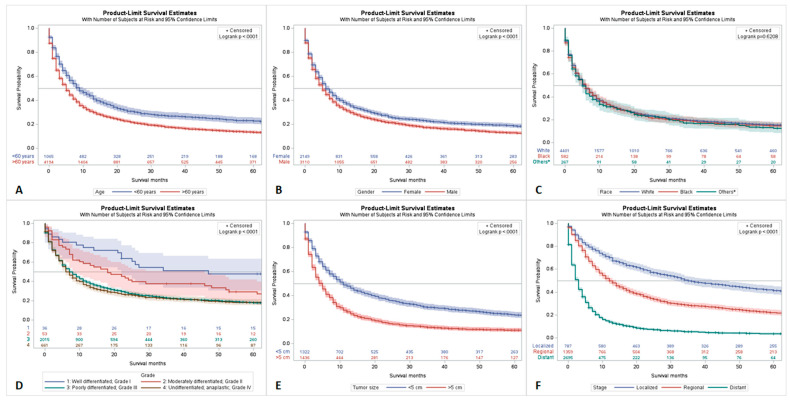
Survival analysis of PSC by Age (**A**), Gender (**B**), Race (Others* = Asian or Pacific Islander, and American Indian or Alaskan) (**C**), Tumor grade (**D**), Tumor size (**E**), and Tumor stage (**F**).

There was a statistically significant difference observed when analyzing outcomes by age. Patients greater than 60 years old had significantly worse 5-year survival rates (*p* < 0.001) (Figure 2A). There was also a significant gender disparity observed in the 5-year survival rates. Females had a 5-year survival of 23.1% (95% CI = 20.9–25.4) and males had a 5-year survival of 17.2% (95% CI = 15.5–19.0) (*p* < 0.001) (Figure 2B).

When analyzing outcomes related to race, white patients had the highest overall 5-year survival at 19.9%. This was followed by black, Asian or Pacific Islander, and American Indian or Alaskan patients at 19.6%, 18.0%, and 9.5%, respectively. However, no statistically significant racial disparities were observed (*p* < 0.6208) (Figure 2C).

When analyzing tumor grade, it was found that well-differentiated (grade I) tumors had better outcomes than grade III or grade IV tumors. Tumors greater than 5 cm in size were observed to have statistically significantly worse outcome than tumors ≤5 cm in size (*p* < 0.001) (Figure 2D,E).

When analyzing tumor stage, each stage had distinct prognostic profiles. The localized stage had statistically significant better outcomes than both regionally and distantly staged tumors. Regionally staged tumors had statistically better outcomes than distantly staged tumors (*p* < 0.001) (Figure 2F and Supplementary Table S4).

### 4.2. Survival Characteristics by Treatment Modality

The 5-year survival for 5259 patients was 15.6% with a 95% confidence interval (95% CI = 14.4–16.9), and the cause-specific survival was 19.7% (95% CI = 18.3–21.1). Patients who underwent chemotherapy, surgery, radiation, and combination therapy had a 5-year survival of 19.9% (95% CI = 17.7–22.2), 41.7% (95% CI = 38.9–44.6), and 19.1% (95% CI = 15.1–23.5), 24.8% (95% CI = 17.6–32.7), respectively.

**Figure 3:**
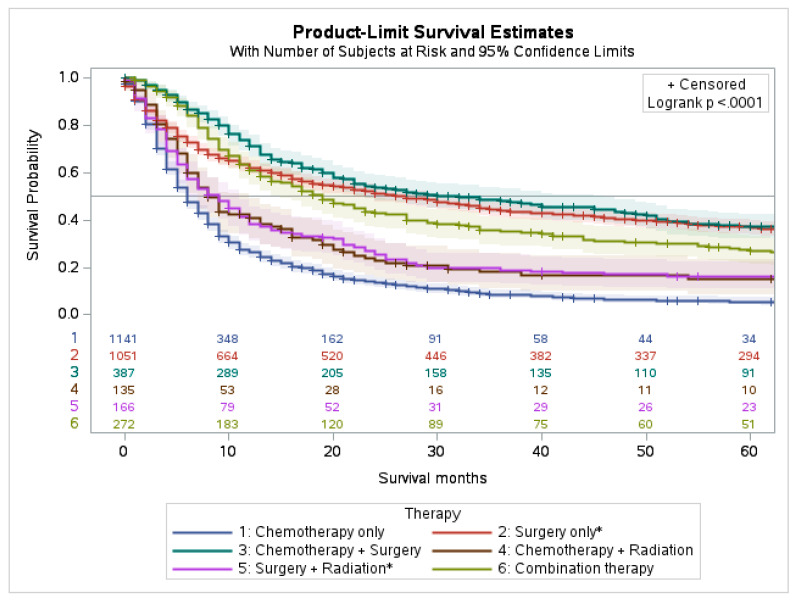
Survival analysis of PSC by different treatment modalities. * The status of chemotherapy is unknown.

Our analysis indicates that highest 5-year survival was observed in patients treated with a combination of chemotherapy and surgery and those treated with surgery only. Patients treated with multimodality therapy (i.e., the combination of chemotherapy with radiation and surgery) also had a favorable survival compared to the other groups. The combination of surgery with radiation, and the combination of chemotherapy with radiation both had similar and slightly worse 5-year survivals. Of all treatment modalities, chemotherapy only had the worst 5-year survival (*p* < 0.001) (Figure 3 and Supplementary Table S3).

### 4.3. Multivariable Analysis

Multivariable analysis through Cox survival regression analysis identified age >60 years (hazard ratio, H.R 1.442, *p* = 0.001), male gender (H.R 1.151, *p* = 0.001), distant stage (H.R 2.934, *p* = 0.001), size > 5 cm (H.R 1.391, *p* = 0.001), bone metastasis (H.R 1.169, *p* = 0.038), brain metastasis (H.R 1.313, *p* = 0.002), and liver metastasis (H.R 1.767, *p* = 0.001) as factors associated with increased mortality (*p*-value < 0.05). Chemotherapy (H.R 0.505, *p* = 0.001) and surgery (H.R 0.382, *p* = 0.001) were factors associated with reduced mortality, (*p*-value < 0.05) (Table 2).

### 4.4. Genetic Mutations in PSC

The genetic mutations data for PSC were extracted from COSMIC (https://cancer.sanger.ac.uk/cosmic, accessed on 25 March 2023) version GRCh37 COSMIC v97. A total of 44,203 cases of lung cancer were evaluated for genetic mutations in the database. In the sub-selection category, all the lung lobes, including right and left lobes, bronchus, and site not specified, were selected for data extraction. In histology selection, all carcinoma cases were selected, which was 43,574 cases. In the sub-histology selection for pulmonary sarcomatoid carcinoma, a total of 298 cases for which genetic analysis was conducted for PSC were identified. The top 20 genes found mutated in PSC were TP53 31% (samples tested = 521), ARID1A 23% (35), NF1 17% (36), SMARCA4 16% (32), KRAS 14% (521), CREBBP 14% (35), MET 13% (402), KMT2A 13% (32), EGFR 11% (511), TSC2 11% (38), ERBB3 11% (37), ARID2 11% (35), TSC1 10% (40), KMT2D 9% (35), STK11 7% (116), PIK3CA 6% (300), NOTCH1 6% (113), NRAS 5% (220), BRAF 3% (340), and MAP2KI 3% (117).

## 5. Discussion

In our analysis, one of the independent factors that affected prognosis and survival was the male gender. We also found that surgery and chemotherapy were associated with improved outcomes, consistent with other population-level analyses [12]. A study that analyzed 151 cases of PSC from 2013 to 2020 showed that brain metastasis negatively impacted overall survival, concordant with the findings of our study [13]. We also found tumor size to be a prognostic factor. This could be explained by a 2018 study that analyzed 114 cases of PSC, where they conveyed that tumor size and proportion of sarcomatoid elements are correlated, so smaller-sized tumors frequently do not contain a sufficient proportion of sarcomatoid elements to impact prognosis [14].

The roles of chemotherapy and radiotherapy in PSC treatment are still being debated since it is widely accepted that PSC displays increased resistance to both compared to other types of NSCLC. The aggressive nature of PSC is partly thought to be due to the increased proportion of poorly differentiated sarcomatous components. Platinum-based doublet chemotherapy was previously a first-line treatment for patients with unresectable PSC in the absence of a targetable driver oncogene. However, the role of chemotherapy is controversial. Some studies show that chemotherapy is a favorable prognostic factor and perioperative chemotherapy reduces recurrence in some patients, whereas other studies suggest that neoadjuvant and adjuvant chemotherapy do not improve survival for those with early stage PSC. The role of radiotherapy in PSC treatment is also controversial [14,15,16]. In our study, we found short-term survival benefits for both chemotherapy and radiotherapy treatments.

### Genetic Profiling and Emerging Therapeutics

The average mutation rate in PSC ranges from 6.9 to 13.6 mutations/Mb [17,18,19]. Various studies have investigated the genomic profiles of PSC. The most commonly mutated genes include *TP53*, *KRAS*, *EGFR*, and *MET*, with variable mutation rates in other genes such as *SMARCA4*, *MLL4*, *NF1*, *PIKCA*, *PTEN*, *NOTCH4*, *APC,* and *TERT* [19]. In a study of 40 biphasic PSC cases, *TP53*, *KRAS*, *PIKCA*, *PTEN*, *STK11*, and *APC* were the most frequently mutated genes [20]. Schrock et al. found *TP53*, *CDKN2A*, *KRAS*, *CDKN2B*, and *NF1* to be the most frequently mutated genes [18]. The variability of mutations is theorized to be driven partly by ethnic heterogeneity, and overlapping mutations suggest a role in PSC pathogenesis. Mutations tend to be enriched in the p53, RTK/RAS, and PI3K pathways specifically. Within the p53 pathway, *TP53* mutation rates ranged from 74 to 79% of PSC patient samples. In one of these studies, 19% of these were truncations which led to inactivation. *TP53* mutant neoplasms also had significantly higher tumor mutation burdens than wild-type *TP53* tumors [17,18,19,21,22]. Within the RTK/RAS pathway, *EGFR*, *KRAS*, *MET*, *BRAF*, *NF1*, and *NRAS* are some genes of interest. In one study, there was an observed 16% mutation rate within *EGFR*, 89% of which were sensitive to available *EGFR* tyrosine kinase inhibitors (TKIs), indicating a potential therapeutic entry point. One mutation, L861Q, resided in a tyrosine kinase domain of *EGFR*, which increases resistance to most *EGFR*-TKIs [17]. Patients with this mutation, however, may be better candidates for afatinib [23,24,25]. In this same patient sample, *MET*, also part of the RTK/RAS pathway, was mutated in 13% of the patients, making them potential candidates for crizotinib, capmatinib, or savolitinib [26,27,28,29]. Another gene of the RTK/RAS pathway, *BRAF*, was mutated in 7% of the patients. One of the *BRAF* mutations, V600E, has been shown to respond well to a combined therapy of dabrafenib and trametinib and has tumor-agnostic regulatory approval [30,31]. The *NF1* gene was mutated in 5% of the patients, all leading to decreased NF1 expression and thus activation of the RTK/RAS pathway. *KRAS* and *NRAS* were mutated in 14% and 4% of the patients, respectively. Moreover, 27% of patients possessed mutations in the PI3K pathway, indicating that PI3K pathway inhibitors may serve as effective treatment options [17,32,33,34]. One specific mutation in the PI3K pathway, E17K, is an oncogenic mutation that could serve as a target for AD2363, a pan-AKT inhibitor [35]. While mutation rates for these gene have differed by sample population, they have been recognized as mutations that play a role in PSC pathogenesis. By better understanding the implicated genes in PSC pathology, clinicians may incorporate this knowledge into their treatment regimen by utilizing more targeted therapy and improving patient outcomes.

Phylogenetic analysis provides strong evidence that the epithelial and sarcomatoid components of PSC arise from a common progenitor and that EMT occurs during the carcinogenesis of PSC [36,37,38]. *TP53*, *KRAS*, and *EGFR* mutations are all early events in EMT and PSC carcinogenesis, suggesting that targeting these genes could inhibit EMT in early PSC and improve outcomes. In a study that molecularly classified PSC, researchers found 195 differentially expressed genes between the epithelial and sarcomatoid components, 36 of which are part of a previously known 76-gene EMT signature. These genes were associated with cell–cell junction, extracellular matrix organization, cell adhesion, and more, indicating that these are all important aspects of cell function that are altered during EMT. Unsupervised clustering of DNA methylation data suggests that epigenetic DNA methylation plays an important role in the regulation and progression of EMT. Thus, these epigenetic modifications may also serve as therapeutic entry points [17,39].

These DNA methylation patterns also provide a basis for molecular classifications. These distinct methylation patterns are divided into three clusters: C1, C2, and C3. These molecular classifications may serve as sources for clinically relevant information by differentiating unique mutations, comorbidities, and molecular mechanisms that are present exclusively or disproportionately in only one of these three clusters. Specifically, we can gain insight on prognostic factors, treatment guidelines, and therapeutic targets. C1 has lower DNA methylation levels than C2 and C3 and is strongly correlated to adenocarcinoma and its sarcomatous components. Most nonsmokers were in C1, and *EGFR* mutations were significantly enriched. *MET* exon 14 skipping and *BRAF* V600E mutations were also identified. Within C1, 50% of patients possessed actionable mutations, suggesting potential for targeted therapy. EMT scores for C3 tends to be lower than C1 and C2. C3 also had significantly longer overall survivability, which may be in accordance with its lower makeup of mesenchymal elements. Most C3 patients were smokers. C2 also had targetable *MET* exon 14 skipping and *BRAF* mutations [17].

Immunotherapy is playing an increasingly important role in the treatment of NSCLC including PSC, commonly being utilized as a first-line therapeutic with or without chemotherapy. In fact, from 2013 to 2016, there were significant decreases in the population mortality of NSCLC, largely attributed to targeted therapeutic advances [40]. Thus, characterization of the immune microenvironment of these clusters is increasingly important. High tumor mutational burden (TMB) and high leukocyte fraction (LF) act as predictive biomarkers for immunotherapy [17,41]. The high TMB and LF in PSC indicate that immunotherapy may serve as a good treatment option for patients with PSC, in accordance with previous dramatic therapeutic responses to immune checkpoint inhibitors in PSC [42,43]. Furthermore, concurrent *TP53/KRAS* mutations may serve as indication that the PSC may respond well to immune checkpoint inhibition [22,44].

PSC displays increased resistance to chemoradiation compared to other NSCLC. This has opened a larger window of opportunity for targeted therapies and immunotherapy, especially given the prevalence of driver mutations and immunogenic profiles, respectively. As more targeted therapy options are being utilized, there is increased emphasis on the genomic profiling of PSC in various ethnically heterogeneous populations to discover driver mutations and elucidate their roles in PSC pathogenesis. With more expansive genomic profiling, there will be increased opportunity for novel targeted therapies. For example, in June 2021, savolitinib, an oral MET tyrosine-kinase inhibitor, was approved for treatment in PSC patients that exhibit the MET exon 14 skipping mutation in China [26,45]. Analysis of the PSC immune microenvironment has also revealed TMB, LF, and the expression of programmed cell death ligand 1 (PD-L1), indicating promising potential for immune checkpoint inhibitors. In fact, anti-PD-1/PD-L1-based therapies are now recommended as first-line treatments for late-stage/metastatic NSCLC regardless of PD-L1 expression. Immunotherapies such as durvalumab, tremelimumab, and nivolumab show promising effects on PSC [45,46,47]. So, while surgical resection is the preferred treatment modality in earlier stages, the effects of chemotherapy and radiotherapy in PSC treatment are being studied. There is growing use and promise of immunotherapy and targeted therapy either as a single agent or in combination with chemotherapy or radiotherapy [11,45].

Previous literature that has examined the clinical characteristics and epidemiology of PSC have examined anywhere from 900 to 1100 cases, whereas our study is the largest cohort with 5200 total PSC cases. Additionally, analysis of tumor size and its effect on prognosis is not as well studied compared to other prognostic factors such as age, gender, stage, or treatment modality. We found tumor size to be a negative prognostic factor, allowing clinicians to better assess patient prognosis. Furthermore, the existing literature examines PSC over a smaller time frame, anywhere from 6 to 12 years, encompassing years as recent as 2016, whereas our analysis encompasses PSC cases over the span of 18 years, including years as recent as 2018 [12,48,49]. This is particularly important as new chemotherapy and radiotherapy regimens, immunotherapy, and targeted gene therapies emerge every year. PSC has shown recent promising therapeutic innovations, highlighting the importance of staying up to date on the epidemiologic trends and clinical data so that recent literature can encompass and reflect the potential upsides gained from emerging therapeutics.

## 6. Limitations

The limitations of this study are due to a lack of completeness of the information received from the SEER database. There were missing data in race, demographics, grading, staging, tumor size, metastasis, and treatment modalities. There were also no data specifying which type of chemotherapy was administered, and the doses of radiation for curative or palliative efforts are not present in the database. Genomic information, family history, environmental exposures, and radiological data are also not available. Additionally, the SEER database cannot account for various factors that determine course of treatment, such as patient preferences, proximity to providers, or comorbidities. This limited the quality of our analysis.

## 7. Conclusions

PSC is a rare type of NSCLC with aggressive clinical behavior. Our study showed that older age (70–79 years), Caucasian ethnicity, male gender, and distant spread was associated with worse clinical outcomes. The combination of surgery and chemotherapy was associated with the best prognosis in comparison to other treatment modalities. While the SEER database does not account for aspects in clinical decision making such as patient preference, proximity to providers, or comorbidities, this analysis provides insight on therapeutic strategies used amongst providers within various stages of disease course and their associated outcomes. We also provide insight on how PSC prevalence and treatment modalities intersect with demographic factors such as age, gender, and race. With this, we aim for our observations to aid clinicians in better stratifying patients for various treatments, in gauging the efficacy of treatments in relation to demographics and tumor characteristics, and in more accurately assessing prognosis. As targeted therapeutics and immunotherapy are emerging as treatment options for PSC, an understanding of how different demographics and patients within various stages of disease respond to existing therapy options is important. As genomic profiling expands, so will targeted therapeutics and the adoption of immunotherapy, and outcomes of PSC should follow a positive direction.

## Figures and Tables

**Table 1:** Demographic profiles and tumor characteristics of 5259 patients with PSC from SEER database, 2000–2018.

Variable (*n* = 5259)		Frequency (%)
Age (years)	1–9	5 (0.1%)
	10–19	2 (0.0%)
	20–29	13 (0.2%)
	30–39	35 (0.7%)
	40–49	255 (4.8%)
	50–59	755 (14.4%)
	60–69	1374 (26.1%)
	70–79	1693 (32.2%)
	80+	1127 (21.4%)
Gender	Female	2149 (40.9%)
	Male	3110 (59.1%)
Race	Unknown	9 (0.2%)
	White	4401 (83.7%)
	Black	582 (11.1%)
	Asian or Pacific Islander	242 (4.6%)
	American Indian or Alaska Native	25 (0.5%)
**Grade (*n* = 5259)**		**Frequency (%)**
Unknown		2494 (47.4%)
Known		2765 (52.6%)
Grade where known (*n* = 2765)		
Well differentiated—Grade I		36 (1.3%)
Moderately differentiated—Grade II		53 (1.9%)
Poorly differentiated—Grade III		2015 (72.9%)
Undifferentiated/Anaplastic—Grade IV		661 (23.9%)
**Variable (*n* = 5259)**		**Frequency (%)**
Stage	Unknown	418 (7.9%)
	Known	4841 (92.1%)
	Stage where known (*n* = 4841)	
	Localized	787 (16.2%)
	Regional	1359 (28.1%)
	Distant	2695 (55.7%)
Size	Unknown	2489 (47.3%)
	Known	2770 (52.7%)
	Size where known (*n* = 2770)	
	In mm or No tumor found	12 (0.4%)
	1–3 cm	616 (22.2%)
	3.1–5 cm	706 (25.5%)
	5.1–7 cm	601 (21.7%)
	≥7.1 cm	835 (30.2%)

**Table 2:** Multivariate analysis of independent factors influencing mortality of 5259 patients with PSC from SEER database, 2000–2018.

Variables		Multivariate Analysis; Hazard Ratio (*p*-Value)
**Age**	>60 years	1.442 (0.001)
**Gender**	Male	1.151 (0.001)
**Stage**	Distant	2.934 (0.001)
**Size**	>5 cm	1.391 (0.001)
**Bone metastasis**	Yes	1.169 (0.038)
**Brain metastasis**	Yes	1.313 (0.002)
**Liver metastasis**	Yes	1.767 (0.001)
**Chemotherapy**	Yes	0.505 (0.001)
**Surgery**	Yes	0.382 (0.001)

## Data Availability

All data are publicly available and will be provided upon appropriate request from the corresponding author.

## Supplementary Materials

The following supporting information can be downloaded at: https://www.mdpi.com/article/10.3390/cancers15092469/s1, Table S1: Lymph node status and metastasis at the time of diagnosis of 5259 patients with pulmonary sarcomatoid carcinoma from the Surveillance, Epidemiology, and End Results (SEER) database, 2000–2018; Table S2: Treatment characteristics of 5259 patients with pulmonary sarcomatoid carcinoma from the Surveillance, Epidemiology, and End Results (SEER) database, 2000–2018; Table S3:Survival data of 5259 patients with Pulmonary sarcomatoid carcinoma from the Surveillance, Epidemiology, and End Results (SEER) database, 2000–2018; Table S4: Survival by race and gender data of 5259 patients with pulmonary sarcomatoid carcinoma from the Surveillance, Epidemiology, and End Results (SEER) Database, 2000–2018.

## Abbreviations

PSC	Pulmonary sarcomatoid carcinoma
NSCLC	Non-small cell lung carcinoma
SEER	Surveillance, Epidemiology, and End Results
COSMIC	Catalogue Of Somatic Mutations in Cancer
EMA	Cytokeratins, epithelial membrane antigen
CEA	Carcinoembryonic antigen
NIH	National Cancer Institute
