# Supplementary material for: Demographics and Clinicopathologic Profile of Pulmonary Sarcomatoid Carcinoma with Survival Analysis and Genomic Landscape
Source: Cancers (Basel). 2023 Apr 26;15(9):2469. doi: 10.3390/cancers15092469 (PMC10177027; doi:10.3390/cancers15092469)
Supplement: Supplementary file 1 [file cancers-15-02469-s001.zip › cancers-2335865-supplementary.pdf]

Demographics and Clinicopathologic Profile of Pulmonary Sarcomatoid Carcinoma with Survival Analysis and Genomic Landscape

Asad Ullah[1], Asim Ahmed[2], Abdul Qahar Khan Yasinzai[3], Kue Tylor Lee[2], Israr Khan[4], Bina Asif[5], Imran Khan[3], Bisma Tareen[3], Saleh Heneidi[6], Jaffar Khan[7], Hina Khan[8], Nabin R. Karki[9], Jaydira Del Rivero[10], Nagla Abdel Karim[11]

Table S1: Lymph node status and metastasis at the time of diagnosis of 5259 patients with pulmonary sarcomatoid carcinoma from the Surveillance, Epidemiology, and End Results (SEER) database, 2000 - 2018.

**Lymph node status (n = 5259)**	
Unknown	3397 (64.6%)
Known	1862 (35.4%)
Lymph node status where known (n = 1862)	
Positive	682 (36.6%)
Negative	1180 (63.4%)
**SEER Metastasis (n = 5259)**	**Frequency (%)**
Unknown	2603(49.5%)
Known	2656 (50.5%)
Where metastasis were known (n = 2656)	
No metastasis	1869 (70.4%)
Bone metastasis only	338 (12.7%)
Brain metastasis only	168 (6.3%)
Liver metastasis only	95 (3.6%)
Bone + Brain metastases	62 (2.3%)
Bone + Liver metastases	74 (2.8%)
Brain + Liver metastases	24 (0.9%)

Combined metastases to bone, brain, and liver	26 (1.0%)

Table S2: Treatment characteristics of 5259 patients with pulmonary sarcomatoid carcinoma from the Surveillance, Epidemiology, and End Results (SEER) database, 2000-2018.

Treatment (n = 5259)	Frequency (%)
Chemotherapy only	1141 (21.7%)
Chemotherapy + Radiation	135 (2.6%)
Chemotherapy + Surgery	387 (7.4%)
Combination therapy (Chemotherapy + Radiation + Surgery)	272 (5.2%)
Chemotherapy unknown | Neither Radiation nor Surgery	1988 (37.8%)
Chemotherapy unknown | Radiation not done | Surgery done	1051 (20.0%)
Chemotherapy unknown | Both Radiation and Surgery done	166 (3.2%)
Chemotherapy unknown | Radiation done | Surgery not done	77 (1.5%)
Both Chemotherapy and Surgery unknown | Radiation not done	28 (0.5%)
Chemotherapy done | Radiation not done | Surgery unknown	12 (0.2%)

Both Chemotherapy and Radiation done | Surgery unknown	2 (0.0%)

Survival analysis by different treatment modalities:

The overall observed 1-, 2-, 3-, 4-, and 5-year survivals were 33.9% (C.I 95%, 32.4-35.4), 23.7% (C.I 95%, 22.3-25.1), 19.5% (C.I 95%, 18.2-20.8), 17.3% (C.I 95%, 16.0-18.6) and 15.6% (C.I 95%, 14.4-16.9), respectively. Cause-specific 1-, 2-, 3-, 4-, and 5-year survivals were 36.4% (C.I 95%, 34.8-38.0), 26.6% (C.I 95%, 25.1-28.1), 22.8% (C.I 95%, 21.3-24.2), 21.0% (C.I 95%, 19.6-22.4) and 19.7% (C.I 95%, 18.3-21.1), respectively. For those treated with chemotherapy, the 1-, 2-, 3-, 4-, and 5-year survivals were 44.1% (C.I 95%, 41.5-46.6), 29.3% (C.I 95%, 26.9-31.8), 24.1% (C.I 95%, 21.8-26.5), 21.7% (C.I 95%, 19.5-24.1) and 19.9% (C.I 95%, 17.7-22.2). For those treated with surgery, the 1-, 2-, 3-, 4-, and 5-year survivals were 66.7% (C.I 95%, 64.0-69.2), 53.1% (C.I 95%, 50.3-55.9), 47.3% (C.I 95%, 44.4-50.0), 44.2% (C.I 95%, 41.4-47.1) and 41.7% (C.I 95%, 38.9-44.6), respectively. For those treated with radiation, the 1-, 2-, 3-, 4-, and 5-year survivals were 48.6% (C.I 95%, 43.8-53.2), 31.4% (C.I 95%, 26.9-36.0), 23.7% (C.I 95%, 19.5-28.2), 20.6% (C.I 95%, 16.6-25.0) and 19.1% (C.I 95%, 15.1-23.5). For those treated with a combination therapy (chemotherapy, surgery, and radiation combined), the 1-, 2-, 3-, 4-, and 5-year survivals were 65.7% (C.I 95%, 57.8-72.5), 42.8% (C.I 95%, 34.7-50.7), 33.3% (C.I 95%, 25.6-41.2), 28.0% (C.I 95%, 17.6-32.7), and 24.8% (C.I 95%, 17.6-32.7). (Table 3).

Table S3: Survival data of 5259 patients with Pulmonary sarcomatoid carcinoma from the Surveillance, Epidemiology, and End Results (SEER) database, 2000 - 2018.

Survival	Overall Observed survival % (C.I. 95%)	Cause specific survival% (C.I. 95%)	Chemotherapy % (C.I. 95%)	Surgery% (C.I. 95%)	Radiation % (C.I. 95%)	Chemo + Surgery + Radiation - Combined treatment

						% (C.I 95%)
1 year	33.9% (32.4-35.4)	36.4% (34.8-38.0)	44.1% (41.5-46.6)	66.7% (64.0-69.2)	48.6% (43.8-53.2)	65.7% (57.8-72.5)
2 years	23.7% (22.3-25.1)	26.6% (25.1-28.1)	29.3% (26.9-31.8)	53.1% (50.3-55.9)	31.4% (26.9-36.0)	42.8% (34.7-50.7)
3 years	19.5% (18.2-20.8)	22.8% (21.3-24.2)	24.1% (21.8-26.5)	47.3% (44.4-50.0)	23.7% (19.5-28.2)	33.3% (25.6-41.2)
4 years	17.3% (16.0-18.6)	21.0% (19.6-22.4)	21.7% (19.5-24.1)	44.2% (41.4-47.1)	20.6% (16.6-25.0)	28.0% (17.6-32.7)
5 years	15.6% (14.4-16.9)	19.7% (18.3-21.1)	19.9% (17.7-22.2)	41.7% (38.9-44.6)	19.1% (15.1-23.5)	24.8% (17.6-32.7)

Survival analysis by gender and race:

The cumulative White race 1-, 2-, 3-, 4- and 5-year survivals were 36.5% (C.I 95%, 34.7-38.2), 26.7% (C.I 95%, 25.0-28.3), 22.9% (C.I 95%, 21.3-24.5), 21.2% (C.I 95%, 19.6-22.7), and 19.9% (C.I 95%, 18.4-21.5), respectively. For Black race, the 1-, 2-, 3-, 4-, and 5-year survivals were 37.8% (C.I 95%, 33.2-42.3), 26.7% (C.I 95%, 22.5-31.0), 23.1% (C.I 95%, 19.0-27.3), 20.5% (C.I 95%, 16.5-24.7), and 19.6% (C.I 95%, 15.7-23.9), respectively. For Asian or Pacific Islander race, the combined 1-, 2-, 3-, 4-, and 5-year survivals were 34.0% (C.I 95%, 27.4-40.7), 26.1% (C.I 95%, 19.9-32.6), 20.8% (C.I 95%, 15.0-27.3), 19.9% (C.I 95%, 14.2-26.4) and 18.0% (C.I 95%, 12.4-24.5). For American Indian or Alaska native race, the combined 1-, 2-, 3-, 4-, and 5-year survivals were 23.8% (C.I 95%, 8.7-43.1), 19.0% (C.I 95%, 5.9-37.7), 14.3% (C.I

95%, 3.6-32.1), 14.3% (C.I 95%, 3.6-32.1) and 9.5% (C.I 95%, 1.6-26.1).

The male gender had 1-, 2-, 3-, 4- and 5-year survival rates of 34.3% (C.I 95%, 32.3-36.3), 24.1% (C.I 95%, 22.3-26.0),20.2% (C.I 95%, 18.4-22.0), 18.5% (C.I 95%, 16.7-20.3), and 17.2% (C.I 95%, 15.5-19.0), respectively. For female gender, the 1-, 2-, 3-, 4-, and 5-year survivals were 39.5% (C.I 95%, 37.0-41.9), 30.0% (C.I 95%, 27.7-32.4), 26.4% (C.I 95%, 24.1-28.8), 24.4% (C.I 95%, 22.2-26.8) and 23.1% (C.I 95%, 20.9-25.4), respectively. (Table 4).

Table S4: Survival by race and gender data of 5259 patients with pulmonary sarcomatoid carcinoma from the Surveillance, Epidemiology, and End Results (SEER) Database, 2000 – 2018

**Survival**	**White% (C.I, 95%)**	**Black% (C.I, 95%)**	**Asian or Pacific Islander% (C.I, 95%)**	**American Indian or Alaska Native% (C.I, 95%)**
1 year	36.5% (34.7-38.2)	37.8% (33.2-42.3)	34.0% (27.4-40.7)	23.8% (8.7-43.1)
2 years	26.7% (25.0-28.3)	26.7% (22.5-31.0)	26.1% (19.9-32.6)	19.0% (5.9-37.7)
3 years	22.9% (21.3-24.5)	23.1% (19.0-27.3)	20.8% (15.0-27.3)	14.3% (3.6-32.1)
4 years	21.2% (19.6-22.7)	20.5% (16.5-24.7)	19.9% (14.2-26.4)	14.3% (3.6-32.1)
5 years	19.9% (18.4-21.5)	19.6% (15.7-23.9)	18.0% (12.4-24.5)	9.5% (1.6-26.1)

**Survival**	**Male%<br>(C.I, 95%)**	**Female%<br>(C.I, 95%)**
1 year	34.3% (32.3-36.3)	39.5% (37.0-41.9)
2 years	24.1% (22.3-26.0)	30.0% (27.7-32.4)
3 years	20.2% (18.4-22.0)	26.4% (24.1-28.8)
4 years	18.5% (16.7-20.3)	24.4% (22.2-26.8)
5 years	17.2% (15.5-19.0)	23.1% (20.9-25.4)
